# Two Excellent Selections

**Published:** 1897-04

**Authors:** 


### TWO EXCELLENT SELECTIONS.

GOVERNOR ROBERT B. SMITH, of Montana, has called Dr. M. E. Knowles to the position of State Veterinarian. Dr. Knowles, who is a graduate of the American Veterinary College and a member of the United States Veterinary Medical Association, is peculiarly well equipped by virtue of a wide and varied practical experience in the veterinary profession, having served in official positions where extensive experience has fitted him for excellent State work. We are sure there will be no backward step in this hustling, aggressive State in caring for its valuable live-stock industries, and there will be an earnest disposition to broaden the field of usefulness and employment of a larger number of veterinarians. Dr. Knowles succeeds Dr. Robert H. Bird, who has held the position for several years.

Franklin, Pa., has chosen for its mayor Dr. George B. Jobson, a veterinarian well known throughout the oil country. The doctor is a member of the Pennsylvania State Veterinary Medical Association, and has not forgotten to combine with his professional duties those of good citizenship. In many ways he bas always shown his interest and zeal in the welfare of his adopted city, and she has now chosen him to preside over her needs and destiny as chief magistrate. We are proud of this recognition of one of our members, and doubly so of his keen interest in good politics, and there is assured to Franklin a wise and broad administration of her public affairs and a keener interest in her well-being through a more wholesome and pure food-supply than she has ever enjoyed. We congratulate our colleagues on the merited recognition accorded them.

---

WHY do certain Western live-stock journals continually inveigh against the bringing of sheep across the Mexican border, as if this was the only source of scab among sheep, when some of the Western States find it necessary to quarantine against all the States around them for the same cause. This savors too much of the actions of foreign countries against our own meat-products.

---

THE Army Legislative Bill has gone to the extra session of Congress. And with better prospects of returning prosperity, through a large balance of trade in our favor, it is felt that the one chief obstacle in its passage, that of need of retrenchment, will not be so forceful, and with the continued aid and support of the profession the committee is sanguine and hopefully confident of its being engrafted upon our national statutes.

Let everyone give a helping hand.

---

THE death of Dr. John R. Hart, of Philadelphia, noted in this number, removes from the profession a valuable member, a conscientious worker, a devoted student of his calling, and a valued citizen of this community.

It would seem that the lower branch of our national government has run mad in its zeal to pander to every locality and interest in rearing up a tariff barrier that will be as surely wiped off our statutes as the rising of the sun. Its intention to place a tariff on books and scientific instruments intended for libraries and institutions of scientific teaching is a fair sample of its general character, and this effort to restrain the attainment of scientific knowledge should arouse a protest from every advocate of higher education.

---

Our May number will be as replete with interesting matter as our present issue. Among the leading features will be the commencement exercises of the various veterinary colleges; much original matter intended for this issue and correspondence, both interesting and important; several important book reviews, and much other matter crowded out of this number.

---

One of the members of Congress from Nebraska proposes, in conjunction with the appropriation bill to the Department of Agriculture, to rescind all the movements and decisions of the past three years that have led to so wise an establishment of the merit system in this department. We ask him to read the signs of the times, to note particularly the selfish interests only that give support to this movement, and, on the other side, balance the strong influence and unselfish advocacy of those who favor its retention. It will require but a moment for a wise man to decide on which side of the fence he should get down, and to bear in mind the experience of the Ohio man who trifled with the heels of the mule, and who afterward was not near so good-looking, but he had an all-fired sight more sense. Don't trifle with a good law, a true evidence of national progress. DON'T MONKEY WITH THE BUZZ-SAW.

---

Prospective changes in the editorial direction and ownership of the *Veterinary Magazine* involve the retirement of Professor Pearson. Professors Adams and Harger will maintain the periodical and assume the chief direction of the same.

